# Epilepsy and coeliac disease in children: a narrative review

**DOI:** 10.3389/fped.2026.1734323

**Published:** 2026-02-09

**Authors:** L. Lonoce, E. Laschi, M. Minerva, F. Lotti, M. R. Curcio, A. Francioni, M. Pacenza, S. Grosso

**Affiliations:** 1 Clinical Pediatrics, Azienda Ospedaliero-Universitaria Senese, Siena, Italy; 2 Clinical Pediatrics, Department of Molecular Medicine and Development, Azienda Ospedaliero-Universitaria Senese, University of Siena, Siena, Italy

**Keywords:** coeliac disease, epilepsy, neurology, pediatric, seizures

## Abstract

**Background:**

Neurological symptoms have been reported as extraintestinal manifestations (EIMs) in 3%–10% of children diagnosed with Coeliac disease (CD). Specifically, the prevalence of epilepsy has been reported to be approximately 1% in pediatric patients with CD, and this population shows an increased risk of developing epilepsy. However, there is still no comprehensive review of epidemiological data, seizure semiology, and electroencephalographic findings of epilepsy in pediatric patients with CD.

**Methods:**

We conducted a narrative review to provide the most recent data regarding prevalence of epilepsy in CD children, describe the typical seizure semiology reported in literature according to the recent classification of International League Against Epilepsy (ILAE) and illustrate the management of epilepsy in this specific population.

**Results:**

Epidemiological data regarding the prevalence of epilepsy in CD children and vice versa are heterogenous due to different inclusion criteria considered in various studies. Overall, an increased risk of epileptic disorder in CD patients has been described in some studies. On the other hand, a higher risk of CD has been reported in children diagnosed with certain types of epileptic disorders [e.g., idiopathic generalized epilepsies]. In terms of seizure semiology, the majority of children with CD present with generalized tonic-clonic seizures or focal seizures, usually of temporal or occipital origin. However, few other patients may experience other types of seizures, such as typical and atypical absences. A subgroup of CD patients presenting with occipital seizures and occipital calcifications on neuroimaging can be diagnosed with CEC syndrome (CD, epilepsy and calcification). A different response to gluten free diet in terms of seizure control has been observed in CD patients.

**Conclusions:**

Clinicians should be aware of this possible neurological EIMs of CD in order to facilitate early recognition and refer patients for further investigations when required.

## 1 Introduction

Coeliac disease (CD) is a systemic immune-mediated disease triggered by gluten ingestion in genetically predisposed patients. The global prevalence of CD has dramatically risen during the last century and it has been estimated that CD may affect from 1% to 1.93% of the population (1–4).

The clinical presentation of CD includes a broad spectrum of manifestations, ranging from typical malabsorption symptoms, to non-specific gastrointestinal symptoms or extra-intestinal manifestations (EIMs) related to neurological, dermatological, endocrine and hematologic systems (5, 6).

Overall, neurological manifestations are reported in 10% to 22.5% of adults diagnosed with CD (7, 8), whereas in children the global prevalence of neurological symptoms is lower, ranging 3%–10% of pediatric patients 9–11). Specifically, epilepsy has been investigated in CD patients over the years, and a correlation between epilepsy and CD has been proved to be bidirectional in both children and adults, with an elevated prevalence of CD reported in epileptic patients and vice versa a higher rate of epileptic manifestation has been noted in CD patients (12).

However, a comprehensive review of all the data regarding the prevalence of epilepsy in CD patients, the most common seizure semiology along with electroencephalographic (EEG) findings in CD children is still missing. Furthermore, studies investigating epilepsy in CD have adopted different classifications of epileptic manifestations over time, resulting in a lack of homogeneity in the description of the clinical and EEG features of epilepsy in patients with CD. Therefore, we aimed to provide a comprehensive overview of the available literature on epilepsy and CD, together with a review of the clinical and electrophysiological manifestations of epilepsy according to the most recent classification of seizures and epileptic syndromes proposed by the International League Against Epilepsy (ILAE).

## 2 Methods

A comprehensive search of literature indexed was conducted on electronic databases MEDLINE (PubMed) and Ovid EMBASE and Cochrane library, combining keywords related to epilepsy (epilepsy, seizures, epileptic syndromes, neurology) with keywords for coeliac disease (coeliac disease, gluten sensitivity) and keyword to select pediatric population (children, pediatric). We included all types of study design (cohort, case-control, case series, case reports etc.) regarding children receiving a diagnosis of CD, according to the criteria by European Society of Pediatric Gastroenterology, Hepatology and Nutrition (ESPGHAN). We exclusively considered data written in the English language. No limitation regarding publication date was placed.

Two investigators independently screened the abstract available in literature (*n* = 144) and reviewed all the potential studies included in the review (*n* = 62). Any discrepancies were resolved through discussion or by a third reviewer, if necessary. Manual backward citations on the basis of references of included studies were performed in order to add further relevant studies. We included a total of 41 articles.

Although this is a narrative review, the study selection has been conducted using predefinite criteria to minimize selection bias.

## 3 Results

### 3.1 Epidemiological data

#### 3.1.1 Prevalence of epilepsy in CD children

The earliest epidemiological data regarding the association between epilepsy and CD come from a small retrospective case control study by Chapman in 1978, which estimated a prevalence of 5.5% of epilepsy in CD patients (adult and children) (12). By contrast, Hanly et al. in 1982, reported a prevalence rate of 1% of epileptic manifestation in a population of 177 CD biopsy-proven patients. In both studies, the diagnosis of epilepsy was defined on the basis of an initial screening through a self-questionnaire completed by CD patients, followed by neurological review and electroencephalographic recording in a selected subgroup of individuals (13). A similar prevalence of 1.1% was confirmed a few years later by Vascotto and Fois on a large multicenter study including 3,969 patients diagnosed with CD (14). These prevalence rates of epilepsy were substantially comparable with those of the general population.

At the beginning of the Twenties, further studies investigating epidemiology of epilepsy in the CD population were published, with controversial results. From one side, Cakir did not find any case of epilepsy in a small group of CD patients (27 patients) who underwent neurological evaluation along with EEG recording (15). Similarly, Ruggieri described a low rate of epilepsy (1.18%) in a group of 835 CD patients undergoing a neurological evaluation and eventually further diagnostic tests (16). Pengiran Tengah confirmed these data in a population of 801 CD individuals (17).

On the other hand, Zelnik reported a significantly higher prevalence of epileptic disorders in children with CD (7.2%), though this retrospective study included a wide range of epileptic manifestations including febrile seizures (18).

The most recent epidemiological statistics has been published in a population-based cohort study by Canova in 2020, which estimated a prevalence of 2.6% of epilepsy in a cohort of 1,215 Italian CD children vs. a rate of 1.3% found in matched healthy controls. This study identified epileptic patients on the basis of hospital discharge letters (diagnosis of epilepsy not including febrile seizures) or prescriptions of more than 2 epileptic drugs (19). On the basis of these data, an increased risk of epilepsy was estimated in CD patients [odds ratio (OR) 2.03 (95% CI: 1.33–3.10)], in particular when considering generalized seizures (adjusted OR: 2.72; 95% CI: 1.24–5.96), but not focal ones (adjusted OR: 1.35; 95% CI: 0.49–3.73) (19).

Similar results had been published in the same period by Ludvigsson et al., who reported a 1.4-fold higher risk of developing epilepsy in both children and adults (20). In this case, epilepsy was defined on the basis of International Classification of Disease code and did not include either status epilepticus or febrile seizures. The authors found that the likelihood of developing epileptic disorders was elevated in CD patients (2.1 fold increase), as confirmed by a systematic review and meta-analysis included in the study (20).

Overall, recent population-based studies suggest a mildly increased risk of epilepsy in children with CD, with reported odds ratio approximately ranging from 1.4 to 2.6. By contrast, smaller cohort and case control studies report highly variable prevalence estimates.

#### 3.1.2 Prevalence of CD in epileptic children

The first data on prevalence rates of CD in epileptic patients was published by Fois et al, who found 9 cases of CD in a large cohort of 783 Italian epileptic children (estimated prevalence 1.1%). In the study, the diagnosis of CD was confirmed by a jejunal biopsy (21). These prevalence percentages are similar to the general population.

However, more recent epidemiological data regarding the prevalence of epilepsy in CD patients showed controversial results.

Indeed, a number of studies on pediatric epileptic patients confirmed prevalence rates of CD comparable to the general population. Giordano reported a prevalence of 1.2% (5 patients up 272) of CD-specific autoantibodies positivity in an Italian cohort of 272 epileptic patients. Unfortunately, no information regarding duodenal biopsies were provided (22). Similarly, Djuric identified only one case of biopsy-proven CD in a cohort of 125 Turkish epileptic children (prevalence rate 0.8%) (23) while Dalgic estimated a prevalence of 1.17% of CD in a group of children undergoing CD screening and subsequent confirmatory biopsy (24).

Conversely, other studies suggest an increased prevalence of CD in epileptic population including both adults and children. Specifically, a study by Emami described a 3.7% prevalence of biopsy-proved CD in a cohort of 108 epileptic individuals (aged 6–64) (25) while Barshiri et al. reported a 6% prevalence of biopsy-proven CD in a group of 113 epileptic patients (adults and children) (26). Pratesi et al. investigated a large cohort of 255 epileptic subjects (119 children, 136 adults) compared to a control group of 4,405 individuals and estimated that the prevalence of CD was 2.3 times higher in epileptic patients than in controls (27).

Notably, some studies investigated the prevalence of CD in children with particular subgroups of epilepsy, with interesting results.

In 2001 Labate reported a prevalence of histological CD of 8% (2/25) in a small group of patients with focal epilepsy (28). In 2014 Isikay found a prevalence of CD of 1% in subjects with idiopathic generalized epilepsies (IGE) in a large cohort of Turkish children, but a subgroup analysis noted a 15.7% CD prevalence in children with self-limited epilepsy with occipital paroxysms (29). Similarly Dai et al. found a higher prevalence of CD among 90 children with occipital lobe epilepsy compared to healthy controls (30).

On the other hand, a study by Essid in 2003, described a prevalence of 8.1% of biopsy- proven CD in a limited group of children with IGE (31) and Ertekin found significantly elevated prevalence rates of both serological and biopsy proven CD (9.1% vs. 15.6% respectively) in a small cohort of pediatric patients affected by IGE (32). A larger retrospective study by Mavroudi confirmed an increased prevalence of CD in IGE patients (2/255) vs. healthy controls (0/280) (33). Of note, a cross sectional study by Vieira et al. confirmed a higher prevalence of CD (3%) in a cohort of 100 patients with IGE although all patients in the study underwent intestinal biopsy and no villous atrophy was found (34).

Recently, Edalatkhah et al. confirmed a percentage of CD of 7.5% (3/40) in a small cohort of Iranian patients with IGE; the authors observed that patients with CD had a later onset of seizures compared to the others without CD (35).

Finally, the prevalence of CD in children with drug-resistant epilepsy (DRE) has been investigated with controversial results. One study did not identify any cases of CD among 70 children with drug-resistant IGE (36), whereas Swartwood et al. reported an elevated prevalence of CD in patients with DRE (37).

Overall, the prevalence of CD in epileptic patients may be comparable to the general population, at least in studies including only the pediatric population (22–24). However some studies suggest that particular subgroups of epileptic patients may experience an increased risk of CD (31–35).

All epidemiological data regarding prevalence of CD in epilepsy and vice versa are summarized in Table 1.

**Table 1:** Summary of studies regarding the epidemiology of epilepsy in CD and vice versa.

Reference	Type of study	Main prevalence result	N. of cases (*n*. male patients)	Sample size (*n*. male patients)	Mean age or interval of age (years)	Inclusion criteria	Outcome
Bashiri et al. (26)	P	6.1%	7 (4)	113 (54)	37.5	Epilepsy	Biopsy- proven CD
Cakir et al. (15)	CS	0%	0	27 (9)	11.22	CD pediatric only	Epilepsy
Chapman et al. (12)	R, CC	5.5%	9 (4)	165 (NA)	40	CD	Epilepsy
Canova et al. (19)	R, CC	2.6%	15 (NA)	1,215 (455)	7.5	CD pediatric only	Epilepsy
Dai et al. (30)	R, CC	2.2%	2 (1)	90 (40)	6.3	CPE pediatric only	Biopsy- proven CD
Dalgic et al. (24)	R, CC	1.17%	8 (3)	170 (95)	9.76	Epilepsy pediatric only	Biopsy- proven CD
Djuric et al. (23)	R, CC	0.8%	1 (0)	125 (53)	10.51	Epilepsy pediatric only	Biopsy- proven CD
Edalatkhah et al. (35)	CS	7.5%	3 (1)	40 (22)	5.29	IGE pediatric only	Biopsy- proven CD
Emami et al. (25)	CS	3.7%	4 (2)	108 (36)	23.44	Epilepsy	Biopsy- proven CD
Erketin et al. (32)	R	9.1%	12 (2)	77 (40)	8.8	IGE pediatric only	Biopsy- proven CD
Essid et al. (31)	R	8.1%	4 (NA)	49 (23)	NA	IGE	Biopsy- proven CD
Fois et al. (21)	R	1%	9 (NA)	783 (NA)	8–18	Epilepsy pediatric only	Biopsy- proven CD
Ghazizadeh Esslami et al. (36)	CS	0%	0	70 (44)	2–12	DRE	CD
Giordano et al. (22)	R CC	1.2%	5 (NA)	272 (153)	0.5–16	Epilepsy pediatric only	Serological CD positivity
Hanly et al. (13)	CS	1%	2 (NA)	177 (NA)	NA	CD	Epilepsy
Isikay and Kocamaz (29)	R, CC	1%	2 (1)	214 (112)	9.3	IGE pediatric only	Biopsy- proven CD
Labate et al. (28)	P	8%	2 (1)	72 (41)	12.6	CPE	
Ludvigsson et al. (20)	R, CC	-	272 (NA)	28,885 (10,892)	41	CD	Epilepsy
Mavroudi et al. (33)	R; CC	1.96%	2 (1)	255 (137)	7.93	IGE pediatric only	Biopsy- proven CD
Pengiran Tegah et al. (17)	R	2.6%	21 (6)	801 (258)	20	CD	Epilepsy
Pratesi et al. (27)	R, CC	0.7%	2 (2)	255 (142)	1–65	Epilepsy	Biopsy- proven CD
Ruggieri et al. (16)	P, O	1.18%	10 pt (NA)	885 (231)	7.8	CD pediatric only	Neurologic Dysfunction (included epilepsy)
Swartwood et al. (37)	R	64%	36 (11)	56 (22)	NA	CD and epilepsy	DRE
Vascotto and Fois (14)	CS	1.1%	46 (NA)	3,969 (NA)	15.5	CD pediatric only	Epilepsy
Vieira et al. (34)	CS	0%	0	1,000 (NA)	10.7	Epilepsy pediatric only	CD
Zelnik et al. (18)	R, CC	7.2%	8 pt (NA)	322 pt (111 pt CD)	NA	CD	Epilepsy

CC, case-control; CPE, childhood partial epilepsy; CS, cross-sectional; DRE, drug resistant epilepsy; IGE, idiopathic generalized epilepsy; P, prospective; R, retrospective,.

### 3.2 Pathogenesis

A bidirectional association between CD and epilepsy has been demonstrated by epidemiological studies.

Despite the pathogenic mechanisms underlying this connection have not yet been clarified, several hypotheses have been proposed over the time, ranging from free radical accumulation with oxidative stress to toxin deposition, malabsorption of vitamin B12, local deposition of anti tissue transglutaminase IgA antibodies (tTG IgA) leading to impaired function of immune complexes, and vasculitis (38, 39).

The theory of nutrients' malabsorption leading to epileptic manifestations was postulated in a case report by Lea in 1995. The authors described epilepsy and cerebral calcification in a CD patient with folate deficit and arose the theory that folate malabsorption could cause epileptic manifestation and cerebral calcifications, as already observed in children with leukemia treated with folate antagonists and in patients having congenital folate deficiency (40).

However, epilepsy has been observed in CD patients despite the presence of malabsorption and other pathogenic hypotheses were considered.

Specifically, a study found a connection between the level of tTG IgA in epileptic patients and the activity of their seizures which led to theorize a possible role of gluten toxicity and gluten-related antibodies in triggering epileptic manifestations (38). Gluten toxicity may lead to impaired capability of intestinal cells in absorbing vitamin B, which may cause alteration of the homocysteine metabolic pathways and subsequent hyperhomocysteinemia (H cy). Ferretti et al. hypothesised that Hcy—frequently observed in CD—might damage the blood-brain barrier, exposing neuronal tissue to all neuro-irritative metabolites, such as homocysteine itself, a neurotoxic excitatory and proconvulsant amino acid. Neurons respond to these stimuli through hyperexcitability, thereby predisposing subjects to EEG anomalies and epilepsy (41). Furthermore, Hcy may lead to persistent endothelial damage causing blood extravasation and subsequent deposition of calcium salts, leading to occipital calcifications described in the so-called CEC syndrome, a well-defined entity characterised by association among CD, epilepsy and bilateral occipital calcification (41).

Additionally, the production of autoantibodies itself may play a pathogenic role in EIMs of coeliac disease. Indeed, different typologies of autoantibodies (e.g., autoantibodies against gangliosides, anti-transglutaminase 6, collagen etc) have been detected in patients with systemic manifestations of CD, leading to the hypothesis that autoimmunity could be directly involved in pathogenesis of these symptoms (42).

In 2008 a novel enzyme of the group of transaminase proteins' family, transglutaminase 6 (TG6), was identified in CD patients with neurological manifestations such as peripheral neuropathy and cerebellar ataxia (43). Subsequent studies demonstrated that TG6 had the same enzymatic activity of transglutaminase 2 (TG2)—the main autoantigen of CD—and it was predominantly expressed in the central nervous system where it was involved in neurogenesis and motor control (44).

It has been hypothesized that CD patients may develop specific autoantibodies targeting TG6 due to a cross reactivity with TG2 and these antibodies may be involved in the pathogenesis of certain typologies of neuronal dysfunction associated with CD (45). Consistently with these theories, the injection of autoantibodies directed against TG6 in mice's brain has been proved to cause deficit in motor coordination (46) and mutation of TG genes have been observed in patients with spinocerebellar ataxia (47). Further studies are still required to define the pathogenic pathways connecting anti-TG6 antibodies with motor symptoms of CD and to investigate a possible role of TG6 in the context of other neurological manifestations of CD such as seizures.

Other studies demonstrated that CD-specific autoantibodies (e.g., antigliadin) may present a cross-reactivity with synapsin, a multifunctional protein involved in forming synaptic vesicles and neurotransmitter release, suggesting the production of these autoantibodies as a possible mechanisms of neurological impairment observed in patients with CD (41, 48). However, a clear link between antibodies anti-synapsin and neurological symptoms of CD is still lacking.

Furthermore, antigliadin antibodies have been also described in CD patients presenting cerebral vasculitis which led to postulate a possible role of circulating immune complexes of gliadin and anti-gliadin antibodies in pathogenesis of neurological symptoms of CD (49).

A comprehensive summary of all the proposed pathogenic mechanisms involved in the association between CD and epilepsy has been provided in Supplementary Figure S1.

Overall, epilepsy could be included in a broader spectrum of neurological manifestations of CD, which range from gluten neuropathy and ataxia to headache and cognitive impairment. To date, several hypotheses have been made to explain the mechanism of the different neurological symptoms in CD but the precise pathology is still unclear (50).

The cross reactivity between tTG IgA and neuronal antibodies may explain some manifestations such as gluten ataxia and peripheral neuropathy while a perivascular inflammation could be the cause of headache or cognitive impairment. Other etiopathogenic theories include abnormal brain perfusion, gut-micro-biota axis alterations and autonomic system involvement (51), but further studies are still required to clarify the pathogenic relevance of each of these hypotheses.

### 3.3 Seizure semiology

A broad spectrum of seizure semiology has been described in CD patients, ranging from focal seizures, mostly with symptoms related to temporal or occipital lobe, to generalised epilepsy, with variable outcome and response to gluten free diet (52).

The majority of studies available in literature describe the presence of generalised tonic-clonic seizures (GTCS) (14, 16, 19, 22–24, 26, 33, 38, 53–56) or focal impaired awareness seizures (14, 16, 19, 22, 29, 38, 52, 54, 55) in patients with CD and epilepsy. Less frequently, focal aware seizures or focal motor seizures were described (33, 34). In contrast, only a few studies have reported other types of seizure semiology, including typical or atypical absence seizures (38, 53) and myoclonic seizures (16).

In terms of specific epileptic syndromes, a few studies describe cases of self-limited epilepsy with centrotemporal spikes (SeLECTS) (16, 23, 33) or other forms of self-limited epilepsy, primarily with occipital discharges (18, 28–30). Only isolated cases of epileptic spasms or other epileptic and developmental encephalopathies, such as Lennox Gastaut syndrome are described among patients with positive CD-serology (16, 27).

Licchetta et al. reported 7 patients with epilepsy onset in pediatric age, all of these presenting focal seizures (3 occipital, 3 temporal and 1 indeterminate focal seizures). Notably one patient received a diagnosis of progressive myoclonic epilepsy (PME) during a long term follow-up and another one was diagnosed with limbic encephalitis (52).

Some of the cited studies also included patients with febrile seizures (FS), either in the form of FS alone or in the context of a genetic epilepsy with febrile seizures plus (GEFS+) (16, 18, 56, 57).

Finally, Hijaz et al. described an interesting case of status epilepticus and encephalopathy as the presenting manifestation of celiac crisis, with good response to steroid therapy in addition to gluten-free diet (58).

Table 2 provides a brief summary of different typologies of seizures reported in studies involving children with CD.

**Table 2:** Summary of studies describing epileptic semeiology in children with CD.

Reference	No. of cases of epilepsy	Type of epilepsy	No. of cases with cerebral calcifications
Arroyo et al. (63)	32	31/32 focal seizures (21 with focal occipital sezures) 1/32 GTCS	32/32
Bashiri et al. (26)	7 pt	4/7 GTCS 3/7 focal impaired awareness sizures	NA
Canova et al. (19)	15 pt	10/15 GTCS 5/15 focal impaired awareness seizures	NA
Crosato and Senter (66)	1/1	1/1 focal occipital seizures	1/1
Dai et al. (30)	2 pt	2/2 focal occipital seizures	None
Dalgiç et al. (24)	8 pt	8/8 GTCS	None
Diaconu et al. (54)	4 pt	2/4 GTCS 2/4 focal impaired awareness seizures	3/4
Djuric et al. (23)	3 pt	1/3 GTCS 2/3 SeLECTS	NA
Fois et al. (67)	1 pt	1/1 focal impaired awareness seizures	1/1
Giordano et al. (22)	2 pt	1/2 GTCS 1/2 focal impaired awareness seizures* (*fetal distress)	None
Gobbi et al. (62)	29 pt	5/29 GTCS 24/29 focal seizures	29/29
Hijaz et al. (58)	1 pt	1/1 status epilepticus	None
Isikiay and Kocamaz (29)	2 pt	2/2 self-limited epilepsy with occipital spikes	NA
Kieslich et al. (53)	3 pt	2/3 GTCS 1/3 absences	None
Labate et al. (28)	2 pt	2/2 self-limited epilepsy with occipital spikes	1/2
Lea et al. (40)	1 pt	1/1 focal impaired awareness seizures	1/1
Licchetta et al. (52)	7 pt	7/7 focal impaired awareness seizures 1/7 limbic encephalitis 1/7 PME	4/7
Magaudda et al. (65)	8 pt	3/8 focal seizures 4/8 focal seizures + GTCS	8/8
Mavroudi et al. (33)	5 pt	3/5 GTCS 1/5 focal impaired awareness seizures 1/5 SeLECTS	None
Piattella et al. (64)	5 pt	5/5 focal seizures	5/5
Pratesi et al. (27)	1 pt	1/1 Lennox Gastaut syndrome	None
Ruggieri et al. (16)	10 pt	2/10 GCTS 2/10 focal impaired awareness seizures 2/10 myoclonic seizures 1/10 self-limited focal epilepsy 1/10 epileptic spasms	None
Sel et al. (38)	4 pt	2/4 GCTS 1/4 absences 1/4 focal impaired awareness seizures	None
Vaknin et al. (56)	6 pt	3/6 GTCS 3/6 FS	NA
Vascotto et al. (14)	9 pt	6/9 focal impaired awareness seizures 2/9 focal impaired awareness seizures + GTCS 1/9 atypical absences + GTCS	3/9
Ventura et al. (55)	2 pt	1/2 focal impaired awareness seizures 1/2 GTCS	2/2
Verd and Amat (57)	5 pt	5/5 FS	NA
Vieira et al. (34)	2 pt	2/2 focal motor seizures	0/2
Zelnik et al. (18)	8 pt	4/8 FS 1/8 self-limited focal epilepsy 1/8 single unprovoked nonfebrile seizure 2/8 chronic epilepsy	1/8

FS, febrile seizures; GTCS, generalized tonic clonic seizures; NA, not available; pt, patient; PME, progressive myoclonic epilepsy; SeLECTS, self-limited epilepsy with centrotemporal spikes.

### 3.4 Electroencephalographic findings in CD patients

In addition, some authors considered the prevalence of EEG findings in patients with CD in the absence of clinical seizures. Işıkay et al. investigated a cohort of 307 CD children and 197 healthy controls, finding a 7.8% prevalence (24/307 patients) of epileptiform discharges (spike/sharp-wave discharges) in the CD group compared to 0.5% prevalence in the control group. Among the 24 cases of CD patients with EEG abnormalities, 8 patients had never experienced any seizure. In this group, the epileptiform abnormalities were characterized by generalized sharp waves or in the occipital and centrotemporal regions. Overall, the frequency of these EEG anomalies was higher in the new diagnosed CD subgroup compared with the patients on a gluten free diet for at least six months (59).

Furthermore, Parisi et al. reported a 47.4% prevalence of abnormal findings at the EEG examinations (focal or generalised sharps and/or spikes and spike-waves) in a small group of 19 CD children without recognizable clinical manifestations. The EEG abnormalities were either diffuse or localized in the frontal, occipital, or parietal regions. These anomalies disappeared after a 6-months GFD in 78% of cases (60).

Similarly, Zochowska-Sobaniec et al. reported a higher prevalence of abnormalities in EEG recordings, including focal sharp waves or slow waves, in 85 CD children compared to the control group; the consumption of gluten by newly diagnosed celiac patients was associated with a high prevalence of EEG abnormalities, while the 6-months or better 12-months GFD showed a positive impact with EEG improvement or normalization (61).

### 3.5 Celiac disease, epilepsy and bilateral occipital calcification (CEC) syndrome

CEC syndrome (Celiac disease, Epilepsy, and bilateral occipital Calcification) is a well-defined clinical picture of both adults and children characterized by the combination of focal seizures usually originating from the occipital lobe, neuroimaging findings of bilateral cortical and subcortical occipital calcification, and CD (62).

In terms of semiology, the majority of children present focal seizures as described in a large case series by Gobbi in 1982 (24 out of 29 of CEC patients with focal seizures) and by Arroyo in 2002 (31 out of 32 of CEC patients) and confirmed by smaller case series by Piattella (62–64). Other patients may present with both focal seizures and GTCS (21, 65, 66). Interestingly, the diagnosis of CD seems to follow the onset of epilepsy in the majority of case series (62, 65) and symptoms of malabsorption may be even absent, with patients undergoing EGDS after diagnostic evaluation for epilepsy (65).

However, the involvement of occipital lobe in CD-associated epilepsy has been demonstrated regardless of presence of calcifications and some evidence suggest that children presenting occipital epilepsy may have an increased risk of CD (31, 32). The reason for this preferential occipital localization in CEC patients is still unclear although it has been hypothesised a selective susceptibility of occipital lobe to immunological phenomena connected with CD (62). Similarly, the pathogenic role of calcification is controversial as they may appear after the onset of epilepsy and their surgical removal seems not to influence the seizure control (52).

In terms of treatment of CEC, it has been noted a positive effect of GFD in seizure control in these patients. Gobbi reported a significant improvement of seizure control in a half of patients treated with GFD, a substantial stability in another half during follow up; the GFD seemed to be more efficient in patients who had been experiencing epilepsy for less years before the diagnosis of CD (62).

A case report by Ventura et al. described a 5-year old patient experiencing occipital focal seizures associated with occipital calcifications who did not respond to conventional antiepileptic drugs (AEDs) until a diagnosis of CD was made and a GFD was started and a supplement with folic acid was started (55).

It seems that the prognosis of epilepsy in CEC depends on a precocious diagnosis and beginning of gluten restriction (52).

### 3.6 Management

In terms of treatment, the effect of GFD on seizure control is variable. In a sizable percentage of patients, adherence to a gluten restriction seems to improve the seizure control allowing weaning dosage or discontinuing one or more AEDs (26). This positive response has been observed in patients presenting with occipital epilepsy (52, 55) Some authors added folic acid supplementation in patients with CD and occipital epilepsy, as the folate deficit has been previously proposed as a possible pathogenic cause of epilepsy in this subgroup of patients (40, 55).

On the other hand, in other studies Essid et al. reported a significant reduction of seizures only in a minority of patients with CD and IGE after the beginning of GFD (31).

A study by Swartwood et al. investigated the risk of DRE in the CD children and adult population with epilepsy. These patients with DRE showed an improvement in seizure control thanks to adherence to GFD (37).

## 4 Discussion

The bidirectional connection between epilepsy and CD has been reported in literature and an increased risk of epilepsy or EEG anomalies has been observed in CD patients compared to the general population (19, 20, 59) in different population-studies.

Significantly, it seems that CD patients tend to experience particular subtypes of epileptic manifestations rather than others. Focal seizures (occipital and temporal seizures) and GTCS are more common while atypical absences and MS are rarer in CD patients. This leads to the consideration that the selected CD population may present a distinct seizure phenotype (31–33). Furthermore, a subgroup of children with CD and focal occipital seizures may also present occipital calcification on magnetic resonance imaging and could be diagnosed with the so-called CEC syndrome which seems to be a distinct clinical entity with different therapeutic management and outcome (52, 62).

In the light of these considerations, some authors wondered whether a target screening of CD should be recommended in patients presenting a particular phenotype of seizures. From one side, a diagnosis of CD in selected epileptic patients may add GFD as another therapeutic option for seizure control, as some studies suggested a beneficial effect of diet on seizure' management (26, 52, 62). On the other hand, other studies failed to prove a significant improvement of epileptic manifestations after GFD (31). Unfortunately, a clear clue to unravel the pathological mechanisms lying behind the neurological manifestations of CD, in particular epilepsy, is still unknown and, consequently, a specific biological explanation of GFD in seizure control is still unclear.

Overall, GFD may be a therapeutic choice for a limited subgroup of patients with CD and particular epilepsy phenotype (i.e., patients with occipital epilepsy) therefore screening CD could be considered in these selected cases.

On the other side of the coin, the association between epilepsy and CD may raise the doubt if CD patients follow up should include a neurological assessment followed by further exams. Currently, epidemiological data could not recommend a routine neurological assessment of these patients but gastroenterologists should be aware of this neurological spectrum of CD manifestations in order to suspect them if required.

Further studies are required to better define the epidemiology of epilepsy in CD patients and vice versa, with a stricter standardization of diagnostic criteria for both pathologies and a shared methodology of enrollment. Similarly, the therapeutic effect of GFD should be clarified with broader prospective and randomized studies which can allow us to draw definite indications regarding the effectiveness of a target screening for CD in a specific group of epileptic patients and the subsequent therapeutic management of these children with epilepsy and CD.

### 4.1 Limitations

The interpretation of data regarding the association between CD and epilepsy should be cautious due to the heterogeneity of the information published in literature.

Specifically, most of epidemiological results derive from different studies in term of design (retrospective, prospective, cross-sectional), sample size, criteria adopted to define epileptic manifestations (chronic epilepsy vs. self-limited epileptic features as febrile seizures) or diagnostic approach to CD (serology vs. biopsy) and enrollment methods (self questionnaire proposed vs. routinary neurological evaluation).

Additionally, data regarding the semiology and therapeutic management of children with CD and epilepsy are provided by case series and case reports with an increased risk of bias.

## 5 Conclusions

Neurological manifestations have been described in CD patients and epilepsy has been reported in several studies, suggesting a potential association between epileptic disorders and CD. A characteristic seizure semiology in CD patients has been suggested and a possible beneficial effect of GFD in seizure control has been observed in specific patient subgroups.

Consequently, it is essential for clinicians involved in the follow up of CD and for neurologists managing patients with epilepsy to be aware of this possible connection in order to suggest further evaluations when required.

## References

[1] Lebwohl B Sanders DS Green PHR. New perspectives on celiac disease. Lancet. (2018) 391(10115):102–14. 10.1016/S0140-6736(17)31796-8 28760445

[2] Choung RS Larson SA Khaleghi S Rubio-Tapia A Ovsyannikova IG King KS Prevalence and morbidity of undiagnosed celiac disease from a community-based study. Gastroenterology. (2017) 152(4):830–839.e5. 10.1053/j.gastro.2016.11.043 27916669 PMC5337129

[3] Rubio-Tapia A Kyle RA Kaplan EL Johnson DR Page W Erdtmann F Increased mortality in adult celiac disease is not related to gluten ingestion: a population-based study. Am J Gastroenterol. (2012) 107(1):145–51. 10.1053/j.gastro.2009.03.059.E22218041

[4] Lionetti E Pjetraj D Gatti S Catassi G Bellantoni A Boffardi M Prevalence and detection rate of celiac disease in Italy: results of a SIGENP multicenter screening in school-age children. Dig Liver Dis. (2023) 55(5):608–13. 10.1016/j.dld.2022.12.023 36682923

[5] Catassi C Verdu EF Bai JC Lionetti E Lionetti E. Celiac disease: an updated overview. Lancet. (2022) 399(10340):2375–86. 10.1016/S0140-6736(22)00794-2 35691302

[6] Husby S Koletzko S Korponay-Szabó IR Mearin ML Phillips A Shamir R European society for pediatric gastroenterology, hepatology, and nutrition guidelines for the diagnosis of celiac disease. J Pediatr Gastroenterol Nutr. (2012) 54(1):136–60. 10.1097/MPG.0b013e31821a23d0 22197856

[7] Hadjivassiliou M Sanders DS Grünewald RA Woodroofe N Boscolo S Aeschlimann D. Gluten sensitivity from gut to brain. Lancet Neurol. (2010) 9(1):103–12. 10.1016/S1474-4422(09)70290-X 20170845

[8] Briani C Zara G Alaedini A Grassivaro F Ruggero S Toffanin E Neurological complications of celiac disease and autoimmune mechanisms: a prospective study. J Neuroimmunol. (2008) 198(1-2):112–6. 10.1016/j.jneuroim.2008.01.008 18343508

[9] Bottaro G Cataldo F Rotolo N Spina M Corazza GR. The clinical pattern of subclinical/silent celiac disease: an analysis on 1026 consecutive cases. Am J Gastroenterol. (1999) 94(3):691–6. 10.1111/j.1572-0241.1999.00938.x 10086653

[10] Lionetti E Francavilla R Pavone P Pavone L Francavilla T Pulvirenti A The neurology of coeliac disease in childhood: what is the evidence? A systematic review and meta-analysis. Dev Med Child Neurol. (2010) 52(8):700–7. 10.1111/j.1469-8749.2010.03647.x 20345955

[11] Nurminen S Kivelä L Huhtala H Kaukinen K Kurppa K. Extraintestinal manifestations were common in children with coeliac disease and were more prevalent in patients with more severe clinical and histological presentation. Acta Paediatr. (2019) 108(4):681–7. 10.1111/apa.14324 29569302

[12] Chapman RW Laidlow JM Colin-Jones D Eade OE Smith CL. Increased prevalence of epilepsy in coeliac disease. Br Med J. (1978) 2(6132):250–1. 10.1136/bmj.2.6132.250 678891 PMC1606375

[13] Hanly JG Stassen W Whelton M Callaghan N. Epilepsy and coeliac disease. J Neurol Neurosurg Psychiatry. (1982) 45(8):729–30. 10.1136/jnnp.45.8.729 7130998 PMC1083165

[14] Vascotto M Fois A. Frequency of epilepsy in celiac disease and vice versa: a collaborative study. In: Gobbi G Ander mann F Naccarato S Banchini G, editors. Epilepsy and Other Neurological Disorders in Coeliac Disease. London: John Libbey (1997). p. 105–11.

[15] Cakir D Tosun A Polat M Celebisoy N Gokben S Aydogdu S Subclinical neurological abnormalities in children with celiac disease receiving a gluten-free diet. J Pediatr Gastroenterol Nutr. (2007) 45(3):366–9. 10.1097/MPG.0b013e31806907e8 17873753

[16] Ruggieri M Incorpora G Polizzi A Parano E Spina M Pavone P. Low prevalence of neurologic and psychiatric manifestations in children with gluten sensitivity. J Pediatr. (2008) 152(2):244–9. 10.1016/j.jpeds.2007.06.042 18206697

[17] Pengiran Tengah DS Holmes GK Wills AJ. The prevalence of epilepsy in patients with celiac disease. Epilepsia. (2004) 45(10):1291–3. 10.1111/j.0013-9580.2004.54104.x 15461685

[18] Zelnik N Pacht A Obeid R Lerner A. Range of neurologic disorders in patients with celiac disease. Pediatrics. (2004) 113:1672–6. 10.1542/peds.113.6.1672 15173490

[19] Canova C Ludvigsson JF Barbiellini Amidei C Zanier L Zingone F. The risk of epilepsy in children with celiac disease: a population-based cohort study. Eur J Neurol. (2020) 27(6):1089–95. 10.1111/ene.14160 31994800

[20] Ludvigsson JF Zingone F Tomson T Ekbom A Ciacci C. Increased risk of epilepsy in biopsy-verified celiac disease: a population-based cohort study. Neurology. (2012) 78(18):1401–7. 10.1212/WNL.0b013e3182544728 22517096

[21] Fois A Vascotto M Di Bartolo RM Di Marco V. Celiac disease and epilepsy in pediatric patients. Childs Nerv Syst. (1994) 10(7):450–4. 10.1007/BF00303610 7842435

[22] Giordano L Valotti M Bosetti A Accorsi P Caimi L Imberti L. Celiac disease-related antibodies in Italian children with epilepsy. Pediatr Neurol. (2009) 41(1):34–6. 10.1016/j.pediatrneurol.2009.02.009 19520271

[23] Djurić Z Nagorni A Jocić-Jakubi B Dimić M Novak M Milićević R Celiac disease prevalence in epileptic children from Serbia. Turk J Pediatr. (2012) 54(3):247–50. 23094534

[24] Dalgiç B Dursun I Serdaroğlu A Dursun A. Latent and potential celiac disease in epileptic Turkish children. J Child Neurol. (2006) 21(1):6–7. 10.1177/08830738060210010301 16551445

[25] Emami MH Taheri H Kohestani S Chitsaz A Etemadifar M Karimi S How frequent is celiac disease among epileptic patients? J Gastrointestin Liver Dis. (2008) 17(4):379–82. 19104696

[26] Bashiri H Afshari D Babaei N Ghadami MR. Celiac disease and epilepsy: the effect of gluten-free diet on seizure control. Adv Clin Exp Med. (2016) 25(4):751–4. 10.17219/acem/43585 27629850

[27] Pratesi R Gandolfi L Martins RC Tauil PL Nobrega YK Teixeira WA. Is the prevalence of celiac disease increased among epileptic patients? Arq Neuropsiquiatr. (2003) 61(2B):330–4. 10.1590/s0004-282(2003000300002 12894262

[28] Labate A Gambardella A Messina D Tammaro S Le Piane E Pirritano D Silent celiac disease in patients with childhood localization-related epilepsies. Epilepsia. (2001) 42(9):1153–5. 10.1046/j.1528-1157.2001.45700.x 11580763

[29] Işıkay S Kocamaz H. Prevalence of celiac disease in children with idiopathic epilepsy in southeast Turkey. Pediatr Neurol. (2014) 50(5):479–81. 10.1016/j.pediatrneurol.2014.01.021 24656466

[30] Dai AI Akcali A Varan C Demiryürek AT. Prevalence of resistant occipital lobe epilepsy associated with celiac disease in children. Childs Nerv Syst. (2014) 30(6):1091–8. 10.1007/s00381-014-2387-6 24566676

[31] Essid M Trabelsi K Jerbi E Boubaker S Gorgi Y Ayed K L'atrophie villositaire au cours de l'epilepsie essentielle [villous atrophy and idiopathic epilepsy]. Tunis Med. (2003) 81(4):270–2. (In French). 12848011

[32] Ertekin V Selimoğlu MA Tan H Konak M. Prevalence of celiac disease in a sample of Turkish children with epilepsy. Pediatr Neurol. (2010) 42(5):380; author reply 380–1. 10.1016/j.pediatrneurol.2010.01.018 20399398

[33] Mavroudi A Xinias I Papastavrou T Karatza E Fotoulaki M Panteliadis C Increased prevalence of silent celiac disease among Greek epileptic children. Pediatr Neurol. (2007) 36(3):165–9. 10.1016/j.pediatrneurol.2006.11.011 17352949

[34] Vieira C Jatobá I Matos M Diniz-Santos D Silva LR. Prevalence of celiac disease in children with epilepsy. Arq Gastroenterol. (2013) 50(4):290–6. 10.1590/S0004-28032013000400010 24474232

[35] Edalatkhah R Aflatooninan M Dehghani Firouzabadi B Fallah R. Evaluation of frequency of celiac disease in children with idiopathic epilepsy. Iran J Child Neurol. (2024) 18(4):61–70. 39478946 10.22037/ijcn.v18i1.36844 PMC11520266

[36] Ghazizadeh Esslami G Allahverdi B Badv RS Heidari M Khosroshahi N Shabani-Mirzaee H Clinical and paraclinical screening for celiac disease in children with intractable epilepsy. Neurol Res Int. (2021) 22(2021):1639745. 10.1155/2021/1639745 PMC8084654 33968447

[37] Swartwood S Wilkes J Bonkowsky JL Trandafir CC. Celiac disease in children: an association with drug-resistant epilepsy. Pediatr Neurol. (2021) 120:12–7. 10.1016/j.pediatrneurol.2021.03.003 33962344

[38] Sel ÇG Aksoy E Aksoy A Yüksel D Özbay F. Neurological manifestations of atypical celiac disease in childhood. Acta Neurol Belg. (2017) 117(3):611–7. 10.1007/s13760-017-0781-z 28434139

[39] Gala D Scharf S Kudlak M Green C Khowaja F Shah M A comprehensive review of the neurological manifestations of celiac disease and its treatment. Diseases. (2022) 10(4):111. 10.3390/diseases10040111 36412605 PMC9680226

[40] Lea ME Harbord M Sage MR. Bilateral occipital calcification associated with celiac disease, folate deficiency, and epilepsy. AJNR Am J Neuroradiol. (1995) 16(7):1498–500. 7484640 PMC8338071

[41] Ferretti A Parisi P Villa MP. The role of hyperhomocysteinemia in neurological features associated with coeliac disease. Med Hypotheses. (2013) 81(4):524–31. 10.1016/j.mehy.2013.06.025 23891042

[42] Yu XB Uhde M Green PH Alaedini A. Autoantibodies in the extraintestinal manifestations of celiac disease. Nutrients. (2018) 10(8):1123. 10.3390/nu10081123 30127251 PMC6115844

[43] Hadjivassiliou M Aeschlimann P Strigun A Sanders DD Woodroofe N Aeschlimann D. Autoantibodies in gluten ataxia recognize a novel neuronal transglutaminase. Br J Dermatol. (2008) 64:332–43. 10.1002/ana.21450 18825674

[44] Stamnaes J Dorum S Fleckenstein B Aeschlimann D Sollid LM. Gluten T cell epitope targeting by TG3 and TG6; implications for dermatitis herpetiformis and gluten ataxia. Amino Acids. (2010) 39(5):1183–91. 10.1007/s00726-010-0554-y 20300788

[45] Thomas H Beck K Adamczyk M Aeschlimann P Langley M Oita RC Transglutaminase 6: a protein associated with central nervous system development and motor function. Amino Acids. (2013) 44(1):161–77. 10.1007/s00726-011-1091-z 21984379 PMC3535377

[46] Boscolo S Lorenzon A Sblattero D Florian F Stebel M Marzari R Anti transglutaminase antibodies cause ataxia in mice. PLoS One. (2010) 5(3):e9698. 10.1371/journal.pone.0009698 20300628 PMC2837746

[47] Wang JL Yang X Xia K Hu ZM Weng L Jin X TGM6 Identified as a novel causative gene of spinocerebellar ataxias using exome sequencing. Brain. (2010) 133:3510–8. 21106500 10.1093/brain/awq323

[48] Alaedini A Green PH Sander HW Hays AP Gamboa ET Fasano A Ganglioside reactive antibodies in the neuropathy associated with celiac disease. J Neuroimmunol. (2002) 127(1-2):145–8. 10.1016/s0165-5728(02)00102-9 12044986

[49] Rush PJ Inman R Bernstein M Carlen P Resch L. Isolated vasculitis of the central nervous system in a patient with celiac disease. Am J Med. (1986) 81(6):1092–4. 10.1016/0002-9343(86)90416-x 3799641

[50] Durazzo M Ferro A Brascugli I Mattivi S Fagoonee S Pellicano R. Extra-intestinal manifestations of celiac disease: what should we know in 2022? J Clin Med. (2022) 11(1):258. 10.3390/jcm11010258 35011999 PMC8746138

[51] Ford RP. The gluten syndrome: a neurological disease. Med Hypotheses. (2009) 73(3):438–40. 10.1016/j.mehy.2009.03.037 19406584

[52] Licchetta L Bisulli F Di Vito L La Morgia C Naldi I Volta U Epilepsy in coeliac disease: not just a matter of calcifications. Neurol Sci. (2011) 32(6):1069–74. 10.1007/s10072-011-0629-x 21630037

[53] Kieslich M Errázuriz G Posselt HG Moeller-Hartmann W Zanella F Boehles H. Brain white-matter lesions in celiac disease: a prospective study of 75 diet-treated patients. Pediatrics. (2001) 108(2):E21. 10.1542/peds.108.2.e21 11483831

[54] Diaconu G Burlea M Grigore I Anton DT Trandafir LM. Celiac disease with neurologic manifestations in children. Rev Med Chir Soc Med Nat Iasi. (2013) 117(1):88–94. 24505898

[55] Ventura A Bouquet F Sartorelli C Barbi E Torre G Tommasini G. Coeliac disease, folk acid deficiency and epilepsy with cerebral calcifications. Acta Pædiatrica. (1991) 80(5):559–62. 10.1111/j.1651-2227.1991.tb11906.x 1908173

[56] Vaknin A Eliakim R Ackerman Z Steiner I. Neurological abnormalities associated with celiac disease. J Neurol. (2004) 251(11):1393–7. 10.1007/s00415-004-0550-9 15592736

[57] Verd S Amat JN. Febrile seizures and celiac disease. J Pediatr. (2008) 153(2):298–9. 10.1016/j.peds.2008.04.034 18639736

[58] Hijaz NM Bracken JM Chandratre SR. Celiac crisis presenting with status epilepticus and encephalopathy. Eur J Pediatr. (2014) 173(12):1561–4. 10.1007/s00431-013-2097-1 23900521

[59] Işıkay S Kocamaz H Sezer S Özkars MY Işıkay N Filik B The frequency of epileptiform discharges in celiac disease. Pediatr Neurol. (2015) 53(1):78–82. 10.1016/j.pediatrneurol.2015.02.006 26092417

[60] Parisi P Pietropaoli N Ferretti A Nenna R Mastrogiorgio G Del Pozzo M Role of the gluten-free diet on neurological-EEG findings and sleep disordered breathing in children with celiac disease. Seizure. (2015) 25:181–3. 10.1016/j.seizure.2014.09.016 25457448

[61] Zochowska-Sobaniec M Jarocka-Cyrta E Lotowska JM Sobaniec P. Effects of a gluten-free diet on brain bioelectrical activity and neurological symptoms in children with celiac disease: a study using EEG assessment. J Clin Med. (2025) 14(3):725. 10.3390/jcm14030725 39941394 PMC11818230

[62] Gobbi G Bouquet F Greco L Lambertini A Tassinari CA Ventura A Coeliac disease, epilepsy, and cerebral calcifications. The Italian working group on coeliac disease and epilepsy. Lancet. (1992) 340(8817):439–43. 10.1016/0140-6736(92)91766-2 1354781

[63] Arroyo HA De Rosa S Ruggieri V de Dàvila MTG Fejerman N. Epilepsy, occipital calcifications, and oligosymptomatic celiac disease in childhood. J Child Neurol. (2002) 17(11):800–6. 10.1177/08830738020170110801 12585717

[64] Piattella L Zamponi N Cardinali C Porfiri L Tavoni MA. Endocranial calcifications, infantile celiac disease, and epilepsy. Child’s Nerv Syst. (1993) 9:172–5. 10.1007/BF00272271 8374923

[65] Magaudda A Dalla Bernardina B De Marco P Sfaello Z Longo M Colamaria V Bilateral occipital calcification, epilepsy and coeliac disease: clinical and neuroimaging features of a new syndrome. J Neurol Neurosurg Psychiatry. (1993) 56(8):885–9. 10.1136/jnnp.56.8.885 8350105 PMC1015143

[66] Crosato F Senter S. Cerebral occipital calcifications in celiac disease. Neuropediatrics. (1992) 23(4):214–7. 10.1055/s-2008-1071345 1407390

[67] Fois A Balestri P Vascotto M Farnetani MA Di Bartolo RM Di Marco V Progressive cerebral calcifications, epilepsy, and celiac disease. Brain De. (1993) 15(1):79–82. 10.1016/0387-7604(93)90011-v 8338215

