# Supplementary material for: Epilepsy and coeliac disease in children: a narrative review
Source: Front Pediatr. 2026 Feb 9;14:1734323. doi: 10.3389/fped.2026.1734323 (PMC12926461; doi:10.3389/fped.2026.1734323)
Supplement: Supplementary file 1 [file Supplementaryfile1.docx]

Supplementary Material

## Supplementary Figures


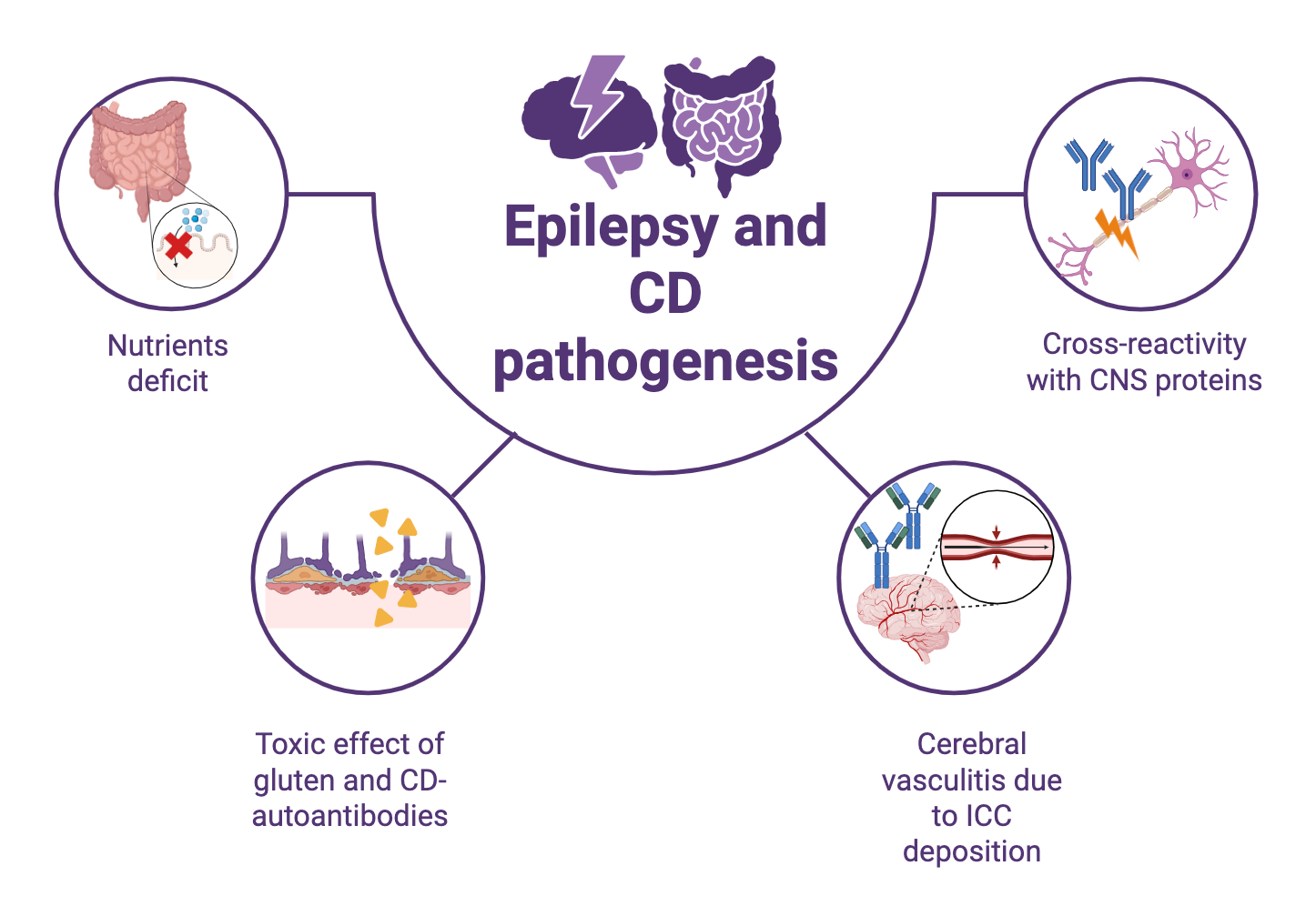


**Supplementary Figure 1.** Possible pathogenic mechanisms of epilepsy in coeliac disease patients. CD: coeliac disease, CNS: central nervous system, ICC: circulating immune complexes
